# Developmental Regulation of Transcription in Touriga Nacional Berries under Deficit Irrigation

**DOI:** 10.3390/plants11060827

**Published:** 2022-03-21

**Authors:** Luísa C. Carvalho, Miguel J. N. Ramos, David Faísca-Silva, David van der Kellen, João C. Fernandes, Ricardo Egipto, Carlos M. Lopes, Sara Amâncio

**Affiliations:** Linking Landscape, Environment, Agriculture and Food Research Centre (LEAF), Associated Laboratory TERRA, Instituto Superior de Agronomia, Universidade de Lisboa, 1649-004 Lisboa, Portugal; miguelramos22@gmail.com (M.J.N.R.); davidfaiscadasilva@gmail.com (D.F.-S.); van-derkellen@hotmail.com (D.v.d.K.); jcfernandes@isa.ulisboa.pt (J.C.F.); ricardo.egipto@iniav.pt (R.E.); cmlopes@isa.ulisboa.pt (C.M.L.); samport@isa.ulisboa.pt (S.A.)

**Keywords:** sustained deficit irrigation vs. regulated deficit irrigation, berry ripening, RNA-Seq, small heat shock proteins, abscisic acid

## Abstract

Grapevine (*Vitis vinifera* L.) is one of the most economically important crops worldwide, especially due to the economic relevance of wine production. Abiotic stress, such as drought, may contribute to low yield, shifts in quality, and important economic loss. The predicted climate change phenomena point to warmer and dryer Mediterranean environmental conditions; as such, it is paramount to study the effects of abiotic stress on grapevine performance. Deficit irrigation systems are applied to optimize water use efficiency without compromising berry quality. In this research, the effect of two deficit irrigation strategies, sustained deficit irrigation (SDI) and regulated deficit irrigation (RDI), in the grape berry were assessed. The effects of different levels of drought were monitored in Touriga Nacional at key stages of berry development (pea size, *véraison*, and full maturation) through RNA-Seq transcriptome analysis and by specific differentially expressed genes (DEGs) monitoring through RT-qPCR. Handy datasets were obtained by bioinformatics analysis of raw RNA-Seq results. The dominant proportion of transcripts was mostly regulated by development, with *véraison* showing more upregulated transcripts. Results showed that primary metabolism is the functional category more severely affected under water stress. Almost all DEGs selected for RT-qPCR were significantly upregulated in full maturation and showed the highest variability at *véraison* and the lowest gene expression values in the pea size stage.

## 1. Introduction

Grapevine (*Vitis* spp.) is a clonal propagated, economically important perennial fruit crop, cultivated worldwide. The grape berry is widely used across the planet, not only in the wine and food industry, but also in the chemical, cosmetic, and agro-industries. In Portugal, this species is represented by a large number of cultivated varieties. According to the International Organization of Vine and Wine (OIV) [1], in 2020 there was a global cultivated area of 7.3 million ha, and in Portugal the vineyard area was 194 thousand ha. In 2020, the global wine production was 260 million hl, with Portugal accounting for 2.4% of the world production (6.4 million hl), ranking 11th worldwide. According to OIV, the volume of wine produced in 2020 decreased significantly from a record high in 2018 (11.5% less), mostly affecting the European Union, with −14.4%. These results were attributed to a combination of unfavorable weather conditions and to an expected drop in demand on the global wine market [1].

The Mediterranean climate is characterized by mild rainy winters, and a hot and dry summer season. Climate change involves higher frequency of extreme events, such as heat waves and drought, leading to scarcer water resources and soil degradation [2,3]. In Portugal, in the summer, the precipitation may be as low as 40% of its current levels by 2050, when compared with data from 1961–1990 [4]. Severe drought in combination with high evaporative demand negatively influences vines’ survival and longevity, and higher temperatures and severe droughts anticipate phenology by several days, while warmer and dryer ripening periods modify berry composition, increasing alcohol contents, and altering the characteristic sensory profiles of wine [5]. The combination of all these changes makes traditional Mediterranean viticulture more difficult, inducing the vinegrowers to adapt to climate change by altering the production systems and choosing different grapevine varieties [6]. 

Plants lack the means to escape from environmental conditions that can induce abiotic stress. As sessile organisms, they have evolved mechanisms at the molecular, cellular, physiological, and developmental levels that enable a quick and efficient response to extreme conditions. For the plant, reproduction is the major issue, but for the producer of an economically valuable crop, the quality of the harvestable organ is of extreme relevance. Concerning grapevine, it has been shown that abiotic stress can influence physiological parameters, fruit composition, and wine quality [5]. These changes can be monitored by scanning the transcriptome of leaves and berries of several varieties [7,8,9]. However, despite the negative impact that drought can have on berry growth and quality [10,11], regulated water deficit has largely been used to restrain vegetative growth and boost berry quality [12].

The grape berry skin provides protection against several microorganisms [13] and against abiotic stress [14] as well. For the wine industry, the interest of the berry skin lies on the accumulation of metabolites, such as acids, anthocyanins, and phenolic compounds (e.g., tannins) that are extremely important for wine quality. Several studies [14,15] showed that the accumulation of secondary metabolites can be influenced by abiotic stress factors. Castellarin et al. [16] showed that under water deficit there is an acceleration in anthocyanin biosynthesis. For these reasons, it is of the utmost importance to understand the different biological mechanisms, and their interactions, that are involved in the response to abiotic signals, in order to improve fruit yield and quality. 

RNA-Seq is a high-throughput sequencing technology that enables the analysis and comparison of whole transcriptomes. RNA-Seq is applied in studies of expression patterns in a specific situation and allows the comparison between different subsets of genes, or the same genes, in different conditions [17]. 

In the present work, the main proposed objective was to analyze the effects of water deficit on global gene expression of the grape berry skin. To achieve this objective, field plants of the Portuguese grape variety Touriga Nacional (TN) were subjected to two deficit irrigation strategies, sustained deficit irrigation (SDI) as the control, and regulated deficit irrigation (RDI) as the more severe water stress treatment. Berry skin from plants in the phenological stages *véraison* (V) and full maturation (M) were used for RNA-Seq analysis. The data obtained by RNA-Seq were validated, and the transcript expression was compared according to the irrigation strategy and the ripening stage. Expression levels of interesting transcripts, some of which were identified in that analysis, were then scanned through RT-qPCR in the skin of berries in three phenological stages, pea size (G), *véraison*, and full maturation, under both treatments. 

## 2. Results and Discussion

The transcript expression in the berry skin of *V. vinifera* var. Touriga Nacional (TN) under two deficit irrigation strategies (SDI and RDI) at *véraison* (V) and full maturation (M) was obtained by RNA-Seq. Reads per kilobase transcript per million (RPKMs) were acquired for all transcripts. The resulting data were analyzed in order to identify candidate genes responding to water stress in the different conditions and developmental stages, thus providing an analysis of the transcriptomic changes in the berry skin of TN plants under deficit irrigation and along berry development. 

### 2.1. Bioinformatics Validation

The correlation between the two biological replicates was assessed through the calculation of the Pearson’s correlation. The correlation coefficient between biological replicates was verified, with values ranging from 0.989 and 0.974 (Table 1). The *p*-value for all samples was lower than the software capacity to calculate such statistical parameter (≤2.5 × 10^−7^). For better visualization, the correlation is represented in a point cloud plot (Figure S1). This strong correlation suggests that the data do not reflect individual deviations but, instead, represent only the irrigation strategy and the developmental stage. Considering that the replicates strongly correlate, the mean values of the biological replicates were used for further analysis.

### 2.2. Revelations from the Whole Transcriptome 

The comprehensive bioinformatics analysis of the RNA-Seq results provided a list of candidate differentially expressed genes (DEGs) that could potentially respond to water stress. From the 55,645 known transcripts in *Vitis*, 15,246 were considered as expressing simultaneously in all samples, according to both filters referred to in Materials and Methods, and 25,429 were never considered active. This indicates that 30% of the transcripts are active on all samples. 

**Figure 1:**
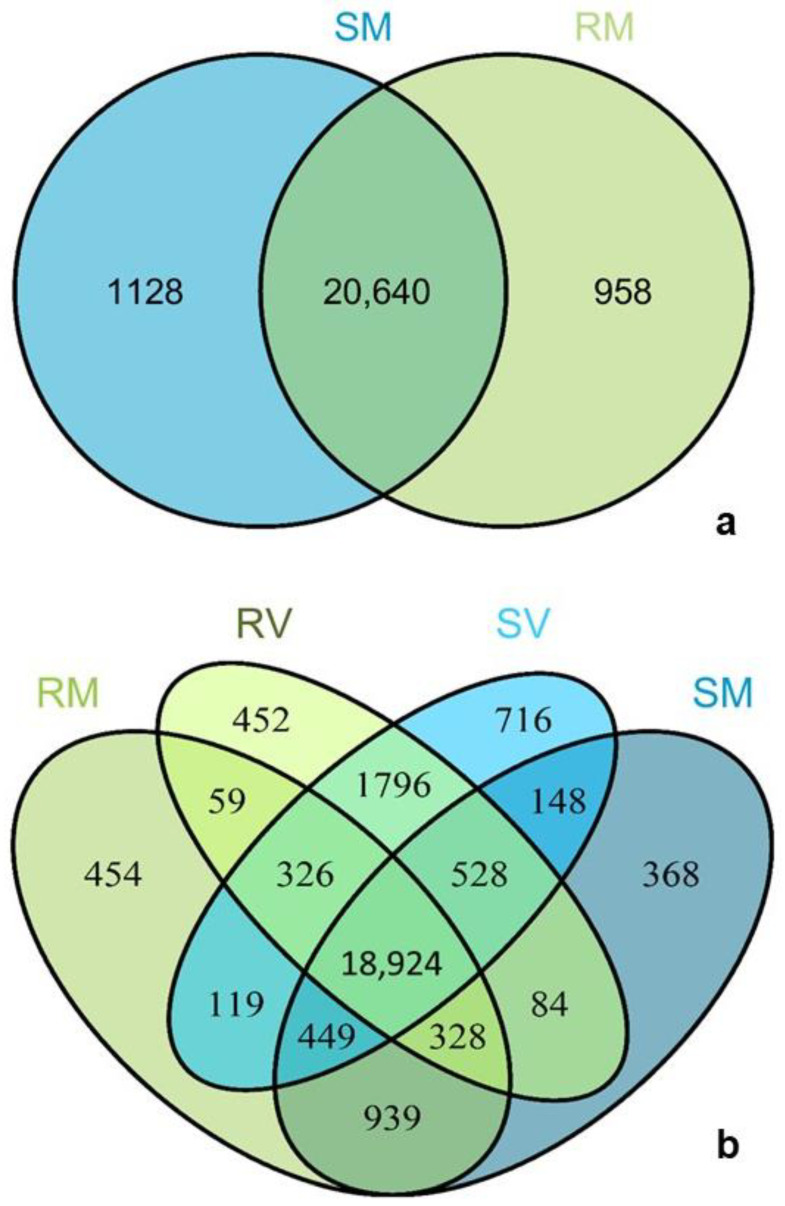
Venn diagrams showing sample-specific transcript analysis considering only the transcripts with length ≥ 150 bp and a threshold of RPKM ≥ 1 for a transcript to be considered as active. (**a**) Full maturation samples under regulated deficit irrigation (RM) and under sustained deficit irrigation (SM); (**b**) samples of regulated deficit irrigation maturation (RM); regulated deficit irrigation *véraison* (RV); sustained deficit irrigation maturation (SM) and sustained deficit irrigation *véraison* (SV).

In the present work, the main difference between the irrigation treatments used is the amount of irrigation, of 36% ETc (crop evapotranspiration) in SDI and 24% ETc in RDI. Consequently, the difference lies within the level of water stress, and not the duration of the periods without irrigation in RDI. To determine the number of genes exclusively expressed in each condition, Venn diagrams were constructed and analyzed. The number of transcripts that were present on multiple or single conditions varies according to the irrigation treatment (Figure 1). In M, 20,640 transcripts were detected in SDI and RDI, with 1128 transcripts exclusively detected in SDI, a number that is higher when compared with RDI (958) (Figure 1a). Comparing the number of transcripts exclusive to RDI in both developmental stages (V and M), it is possible to observe that these are very similar in M (454) and V (452) (Figure 1b). In SDI, there are 716 transcripts unique to V, almost twice the number of transcripts exclusively present in M (368) (Figure 1b). These results indicate a marked difference between irrigation strategies at V, with SDI showing more significantly regulated genes, and are in accordance with the notion that the physiological responses of Touriga Nacional berries to abiotic stress are more evident at *véraison* [12].

**Figure 2:**
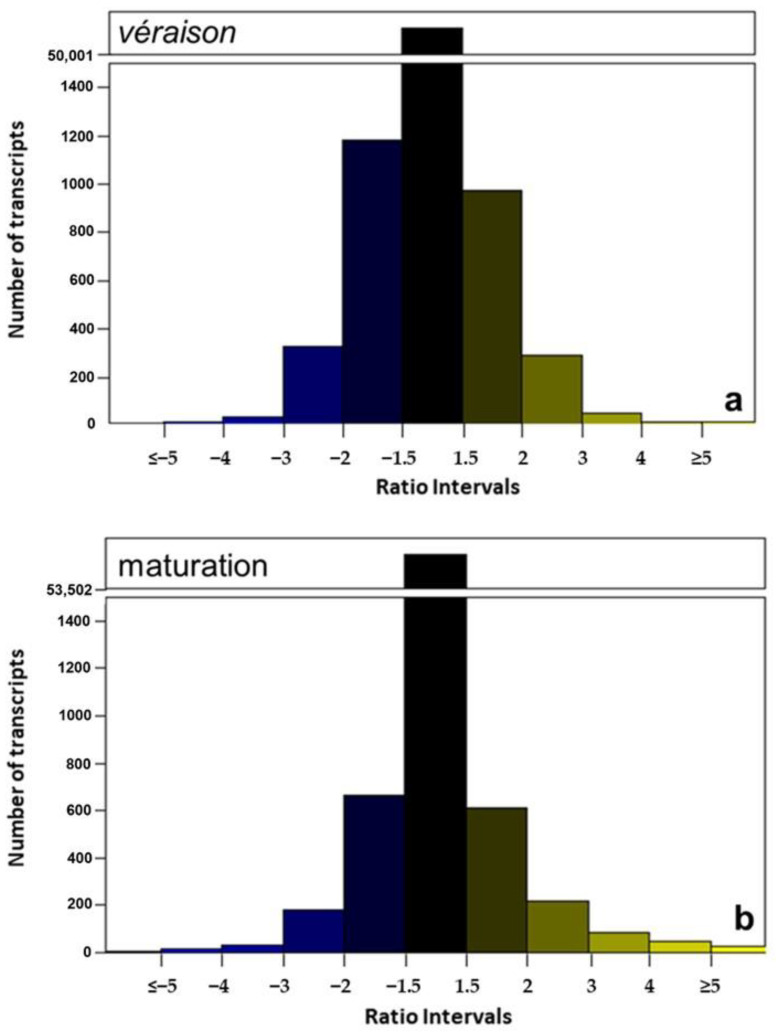
Number of transcripts distribution per ratio interval regulated deficit irrigation/sustained deficit irrigation (RDI/SDI). A scale break was applied from ≈1500 to ≈50,000 transcripts. (**a**) *Véraison* samples and (**b**) maturation samples.

To evaluate the level of response to the irrigation treatments, the expression ratio was calculated, considering as control the treatment with higher water supply (SDI). In order to visualize the transcript ratio distribution, bar plots with the ratio intervals were made. The majority of transcripts (>50,000) fell within the interval between −1.5 and 1.5 ratio, on all samples (Figure 2). The transcripts on this interval are those not considered significantly different between treatments (see Material and Methods), and therefore are not being regulated by water deficit (Figure 2). Approximately 30 transcripts had extreme values of expression ratios (ratio ≤ −5 or ≥5) (Figure 2), indicating that the expression of the transcripts in these intervals is being significantly modulated by water stress. The V sample has a smaller number of upregulated transcripts (13) than the M sample, which falls in line with the strong developmental downregulation of gene expression on this developmental stage [18], while on maturation, a response to stress was observed. This pattern of transcript distribution highlights which transcripts were more significantly regulated.

### 2.3. Functional Profiling of Transcripts 

**Figure 3:**
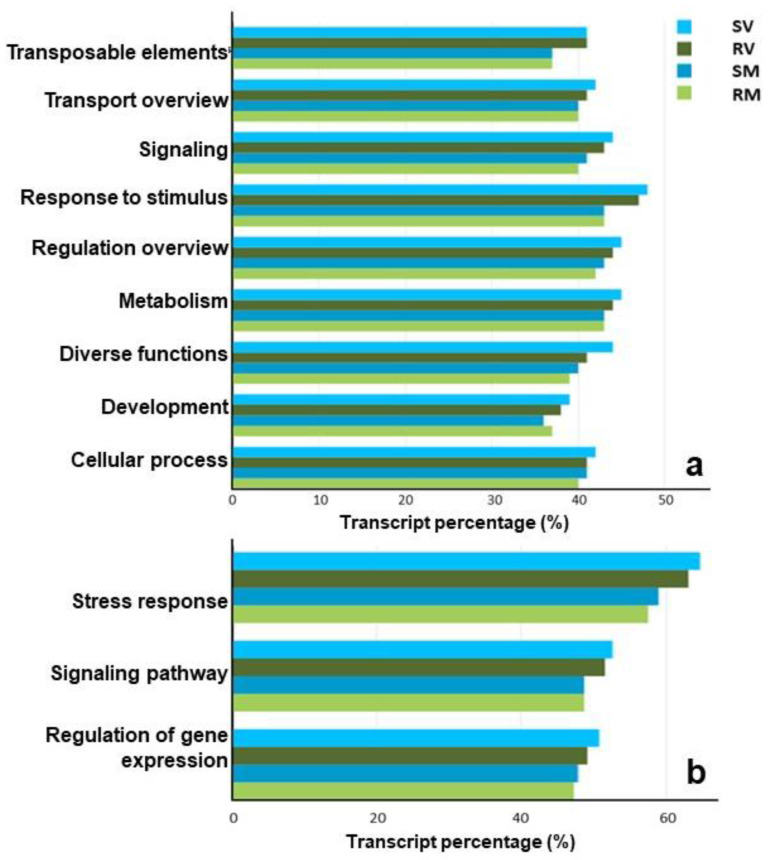
(**a**) Functional profiling of global gene expression for each RNA-Seq library, according to each transcript functional category. The number of active transcripts (length ≥ 150 and RPKM ≥ 1) per functional category was divided by the total transcripts from that category in order to normalize the expression profiles according to the number of transcripts on each class. The transcripts with no class defined in the reference transcriptome (CRIBI Grape genome) are represented in Table 2. (**b**) Sub-functional categories of “response to stimulus”. SV: Sustained deficit irrigation *véraison*; RV: regulated deficit irrigation *véraison*; SM: sustained deficit irrigation full maturation; RM: regulated deficit irrigation full maturation.

To assign the transcripts to functional categories, a plot with expressed isoforms per functional category was organized (Figure 3). This analysis provided a comparison of the transcript percentage between the treatments and developmental stages. In almost every functional category, V had higher percentage of transcripts (Figure 3).

Functional profiling of differentially expressed genes (DEG) expression also brought to light the fact that non-annotated transcript categories still account for a large part of the significantly regulated transcripts (Figure S2, Table 2). In fact, the sum of all unknown, unclear, unclassified, no name, and no hit categories, representing all the transcripts with functions yet to be assigned, is the category with the highest number of transcripts (Table 2). This feature was reported several years ago in the analysis of the 14 K grapevine microarray chip [19] but is still a problem with state of the art technologies, such as RNA-Seq. It reveals that despite the efforts in sequencing and annotating the grapevine genome, many transcripts still have an unspecified function, and some of them correspond to actually relevant stress-responding genes [9]. This indicates that annotation studies are still necessary to determine the functions of these regulated transcripts, and their potential roles on water stress response.

### 2.4. Correlation of Significantly Regulated DEGs with Leaf Water Potential

To verify to what extent the evaluated changes in expression were influenced by the actual level of water stress, a correlation between the amplitude of pre-dawn leaf water potential (Ψ_pd_), quantified in each irrigation regime, and gene expression was performed. The levels of 4794 transcripts showed a strong regression coefficient with Ψ_pd_ measured in the field (R^2^ ≥ 0.90), and 53 transcripts showed an even stronger regression coefficient (R^2^ ≥ 0.99) (Table S1). These linear regressions confirm that changes in the irrigation strategy are directly related with the level of DEG expression. DEGs such as nitrite reductase (VIT_203s0063g00370.1) and a protein involved in cell wall cellulose biosynthesis and deposition (VIT_202s0025g01200.2) were correlated with water stress (Table S1). In addition, a probable galacturonosyltransferase-9 (VIT_214s0066g02350.1) was highly correlated with water stress (R^2^ = 0.90 and ratio = 6.61) (Table S1). Most of the DEGs that correlated with Ψ_pd_ belonged to the functional category metabolism (Table S1). The expression of these transcripts could have an effect on metabolic pathways, therefore leading to changes in the berry skin under water stress. These results, combined with other studies [9,12], provide an indication of the effect of water stress in grapevine transcript modulation.

Some DEGs were downregulated, such as VIT_209s0070g00480.1 (extension-like protein), but most had increases in expression. Two gypsy-like retrotransposons also correlate with Ψ_pd_ (VIT_202s0025g04280.1 and VIT_204s0043g00330.1) (both R^2^ = 0.99) (Table S1). It is known that gypsy-like retrotransposons are well represented in grapevine’s stress-responsive transcriptome [9], but it is still not known how, nor why, their activity is regulated by water stress. Further studies will be necessary to understand the role of these mobile elements in drought response. 

### 2.5. DEG Functional Profiling

#### 2.5.1. Response to Water Stress

**Figure 4:**
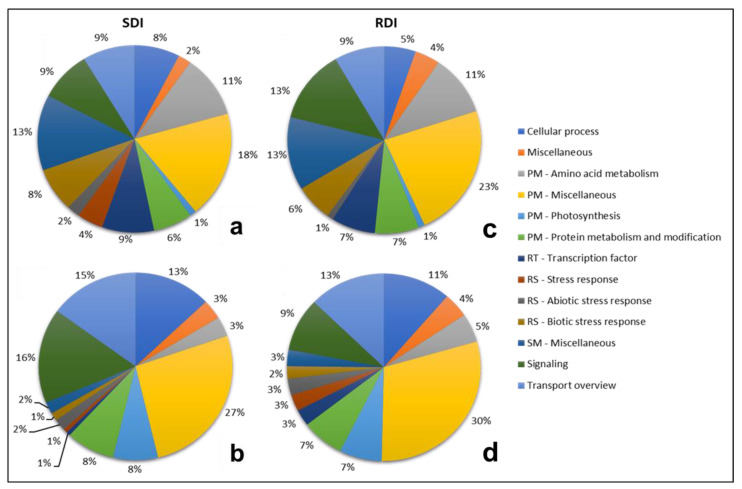
Functional category analysis of significantly regulated DEGs in sustained deficit irrigation (SDI) and regulated deficit irrigation (RDI). Gene expression values were obtained through RNA-seq analysis, and the genes included in these pie charts showed significant differences in expression in SDI and RDI when comparing maturation with *véraison*. Charts (**a**,**c**)—upregulated DEGs; (**b**,**d**)—downregulated DEGs. PM—primary metabolism; RT—regulation of transcription; RS—response to stress; SM—secondary metabolism.

Water stress can affect the expression of genes assigned to several functional categories. Transcriptional changes correlated with the treatment (RDI vs. SDI), and with berry developmental stages (V vs. M), were assessed. Several sub categories of primary metabolism (PM) accounted for a considerable part of the changes (Figure 4), with the functional category “PM—miscellaneous” as the most represented in SDI and RDI, either being up- or downregulated. “cellular process”, “PM—photosynthesis”, and “transport overview” were mostly downregulated, in both conditions. The category “PM—amino acid metabolism” was equally represented as upregulated in SDI and RDI and was almost equally represented as downregulated. In all four pie-charts, “PM—protein metabolism and modification” was more or less equally represented. One of the main reasons for this overrepresentation of PM is that this category includes several basic and life-sustaining functional categories such as carbohydrate, lipid, organic acid, nitrogen and sulfur metabolisms, and carbon fixation, among others. From this group of diverse categories, carbohydrate metabolism tends to be specifically targeted for downregulation. This is not a surprise because the shut-down of basic metabolism is typical in stress conditions [20,21,22].

Genes responsible for response to stress (RS), included in the categories “RS—biotic stress response”, “RS—abiotic stress response”, and “RS—stress response” (both biotic and abiotic) were well represented in SDI and RDI and were predominantly upregulated. “Miscellaneous” showed low representability in all situations and “secondary metabolism (SM)—miscellaneous” was more upregulated in both treatments. The functional categories “signaling”, which includes phytohormones, and “regulation of transcription (RT)—transcription factors” are analyzed in more detail in Table 3. The sub-category “pathway” within the functional category “signaling” comprises four other sub-categories, “calcium sensors and signaling”, “protein kinase”, “protein phosphatase”, and “signaling molecules”, and, for this reason, it had the highest number of entries, being the most represented in SDI downregulated. Overall, “signaling” had a higher representability than “transcription factors”. 

Phytohormones are essential for the ability of plants to adapt to abiotic stresses by mediating a wide range of adaptive responses [23,24]. In this study, “auxin” and “ethylene” were the only two represented in all situations (Table 3). Auxin metabolism was highly downregulated, when comparing to other hormones, in both RDI and SDI. This could be due to the fact that downregulation of auxins may improve the tolerance to drought, as suggested by Zhang et al. [25], who reported that downregulation of Indole-3-acetic acid (IAA) concentration facilitates the accumulation of late embryogenesis abundant proteins (LEAs), which in turn improves drought tolerance. This improvement occurs because LEAs may function as protective macromolecules in detoxification and the alleviation of cellular damage during dehydration [26]. The decrease in IAA may also improve drought tolerance since it may facilitate the promotive action of abscisic acid (ABA) in stomatal closure which, in turn, reduces water loss through transpiration [27]. Under drought stress, ethylene was moderately downregulated when compared to other hormones in RDI and SDI, which is in accordance with previous studies [28]. In SDI, “ABA” and “cytokinin” were moderately downregulated, while “brassinosteroid” was moderately upregulated. “Gibberellic acid” was downregulated in SDI and upregulated in RDI. “Jasmonate salicylate” was upregulated in both SDI and RDI treatments. 

Transcriptional control of the expression of stress-responsive genes is a crucial part of the plant response to a range of abiotic and biotic stresses. Transcription factors (TFs) from several families, such as bZIP, MYB, NAC, and zinc finger, were differentially regulated by drought stress (Table 3). These TFs act in the regulation of several genes related to developmental processes and stress response. The only *bZIP* significantly regulated was downregulated in RDI, which is unexpected since bZIP transcription factors have been shown to enhance tolerance to dehydration and long-term water stress through the synthesis of more protective compounds, such as LEAs and polyamines [29]. Studies focusing on gene expression showed that zinc fingers such as C2H2 and C3HC3 were induced under several types of abiotic stress including drought [30,31].

#### 2.5.2. Differential Regulation between *Véraison* and Maturation

**Figure 5:**
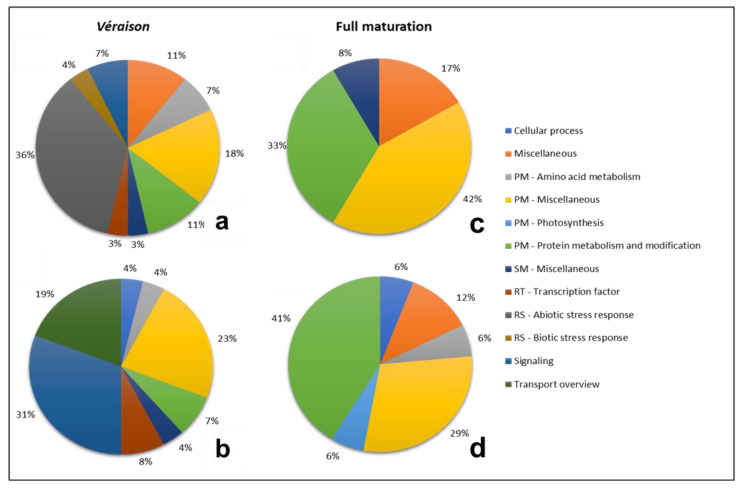
Functional category analysis of significantly regulated DEGs in the developmental stages *véraison* and full maturation. Gene expression values were obtained through RNA-Seq analysis and the DEGs included in these pie charts showed significant differences in expression at *véraison* and full maturation when comparing sustained deficit irrigation (SDI) and regulated deficit irrigation (RDI). Charts (**a**,**c**)—upregulated DEGs; (**b**,**d**)—downregulated DEGs. PM—primary metabolism; RT—regulation of transcription; RS—response to stress; SM—secondary metabolism.

The analysis of the main functional categories in *véraison* and full maturation (Figure 5) revealed clear differences in the modulation of transcription between these developmental stages. Although being the most represented category in Figure 4, “PM—miscellaneous” only showed the same tendency in upregulated DEGs in M. In V, “RS—abiotic stress response” was the mostly upregulated functional category, and none of its genes was significantly downregulated. “Transport overview” showed the opposite pattern, with no upregulated elements and with the highest representability as downregulated. These two categories, alongside with “RS—biotic stress response” and “signaling”, were absent in M. Conversely, “PM—protein metabolism and modification” was more represented in M than in V. “Miscellaneous” was present in all scenarios, except on downregulated DEGs in V. “Cellular process”, “PM—amino acid metabolism”, “PM—photosynthesis”, and “RT—transcription factor”, when present, had a low number of entries. Looking into both “signaling” and “RT—transcription factor” functional categories in detail (Table 4), it was possible to notice that, besides the low amount of entries (much lower than on the treatment comparison, Table 3), there were no sub-categories with significantly regulated DEGs in maturation.

### 2.6. Close-Up on Relevant DEG Modulation and Its Significance

Significantly regulated DEGs in V and M in RDI, as compared to SDI, were ranked by expression levels, and the first ranking ten in each sample were analyzed in detail. Most of them fall within the functional category “metabolism” (Table S2). In fact, in maturation, only one upregulated DEG, annotated on CRIBI as an early-responsive to dehydration stress protein (VIT_202s0109g00230.5), belongs to a different functional category, “response to stimulus”. The ten most downregulated DEGs in M provided an interesting insight into how this variety, in this developmental stage, behaves. Two of the most significantly downregulated DEGs (VIT_212s0035g01910.1 and VIT_218s0089g01270.1) encode HSPs (ratios of −103 and −13, respectively) (Table S2), a family of genes activated to cope with environmental stress [32,33]. The sHSPs reported here are two *HSP22*, which play structural roles in preserving the integrity of cell membranes during stress and are known to return to basal levels shortly after the end of the applied stress [34]. However, in the current work, they were downregulated under severe water stress (RDI) in M. Touriga Nacional is a variety that is tolerant to abiotic stress [35], and an absence of the widely described tendency of upregulation of *sHSPs* upon the onset of stress was already reported in this variety when subjected to heat stress [20].

In V, an interesting upregulated DEG was *CHIA*, encoding a chitinase (VIT_215s0046g01590.2) involved in plant responses to fungal attacks and acting in the cell wall [36]. Another DEG of interest was a *NAC transcription factor* (VIT_215s0048g02270.9), from a plant-specific family, associated with roles in plant resistance including abiotic and biotic stress responses [37]. Also upregulated in V was a *Ca^2+^-transporting ATPase* (VIT_207s0129g00180.2, *CRP*), involved in signaling, that has been implied in drought tolerance in the sweet potato somatic hybrid KT1 [38]. Unexpectedly, one of the most significantly downregulated DEGs in V is associated with drought response (VIT_202s0109g00230.2), annotated on CRIBI as an early-responsive to dehydration stress protein.

**Figure 6:**
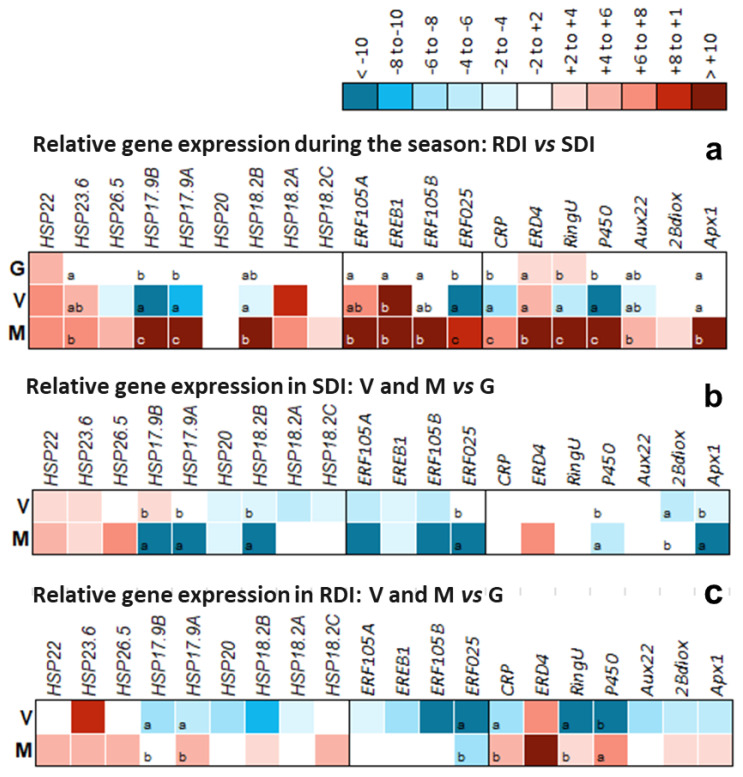
Relative gene expression ratios obtained by RT-qPCR of the DEGs of interest (*HSP22*; *HSP23.6*; *HSP17.9B*; *HSP17.9A*; *HSP20*; *HSP18.2B*; *HSP18.2A*; *HSP18.2C*; *ERF105A*; *EREB1*; *ERF105B*; *ERF025-like*; *CRP*; *ERD4*; *RINGU*; *P450*; *AUX22*; *2ßDIOX1*; *APX1*) in (**a**) regulated deficit irrigation (RDI) when comparing to sustained deficit irrigation (SDI; as control) in pea size (G), *véraison* (V), and full maturation (M); (**b**) in SDI, when comparing G (as control) with V and M; (**c**) in RDI, when comparing G (as control) with V and M. RT-qPCR values were standardized with the CT values of three reference genes; Actin 2; TIF, and TIF-GTP. Different letters indicate significant differences (*p*-value < 0.05) in the expression of each DEG between the development stages. Values within |log_2_ (gene expression level)| < 2 are not significantly different from the respective controls (RDI vs. SDI in (**a**); G vs. V and M in (**b**,**c**)).

For a more detailed and focused analysis in three key development stages, pea size (G), V, and M, using RT-qPCR, DEGs were chosen according to their expression in the RNA-Seq experiment (Table 3, Table 4 and Table S2). As two *sHSPs* were among the ten most significantly regulated DEGS, they were included, together with seven other already identified as responding to abiotic stress in TN plants [8,9,20]. The same was true for *ERFs* (Table 3 and [9]). The other selected DEGs (*CRP*, *RINGU*, *APX1*, *AUX22*, *P450*, *ERD4*, *2βDIOX1*) were significantly expressed in the current RNA-Seq analysis and fall within functional categories of interest, such as hormones (ethylene, auxins, and gibberellins), signaling, and regulation. Figure 6a compares gene expression in RDI using SDI as control in the three developmental stages, while Figure 6b,c compare gene expression in RDI and in SDI during development, using the stage pea size as control.

When analyzing the DEGs response to drought in the different developmental stages (Figure 6a), it was possible to notice that all DEGs were significantly upregulated in maturation, except for *HSP20*, without significant changes in expression. In *véraison,* nine genes were significantly downregulated and seven were significantly upregulated. The pea size stage had the lowest levels of response to drought with only two DEGs significantly upregulated (*HSP22* and *ERD4*), a clear indication that water stress was not yet influencing plant response at that early stage. In fact, pre-dawn leaf water potential (Ψ_pd_) in RDI was *circa* −0.4 MPa (Figure S3), a value consistent with a very mild stress for this variety [35]. *HSP17.9A, HSP17.9B,* and *HSP18.2B* showed significant changes in expression values from one developmental stage to another. *HSP17.9B* showed the lowest gene expression value in *véraison* in comparison to all other genes and developmental stages. The gene family *sHSP* not only responds to heat stress, but also to other abiotic stresses that cause an oxidative response, such as water stress [39]. These proteins confer tolerance to stress through the preservation of the cell membrane integrity, and it is known that, after the end of the stress, they return to basal levels [20,34]. For these reasons, we expected that the sHSPs studied would be upregulated, which is in accordance with data obtained in leaves of TN under heat stress [20].

Regarding TFs within the *ethylene responsive factors* (*ERFs*) family, the four DEGs studied had significant changes, with *ERF025-like* showing the same pattern as the *sHSPs* indicated above, downregulated in *véraison* but upregulated in maturation. These transcription factors can confer stress tolerance through several mechanisms, such as induced expression of genes associated with stress resistance, for example, *early response to dehydration*, *ERD* [40], hormone cross talk [41], and ROS (reactive oxygen species) responsive gene expression in Arabidopsis [37,42,43,44]. Therefore, the upregulation of *ERFs* and of *ERD4* under water stress was expected. Through a cytoscape network analysis of the *ERFs* studied through RT-qPCR, it was possible to verify that these TFs are closely related in the signaling pathway, which suggests that there are interactions between them during the stress response. 

**Figure 7:**
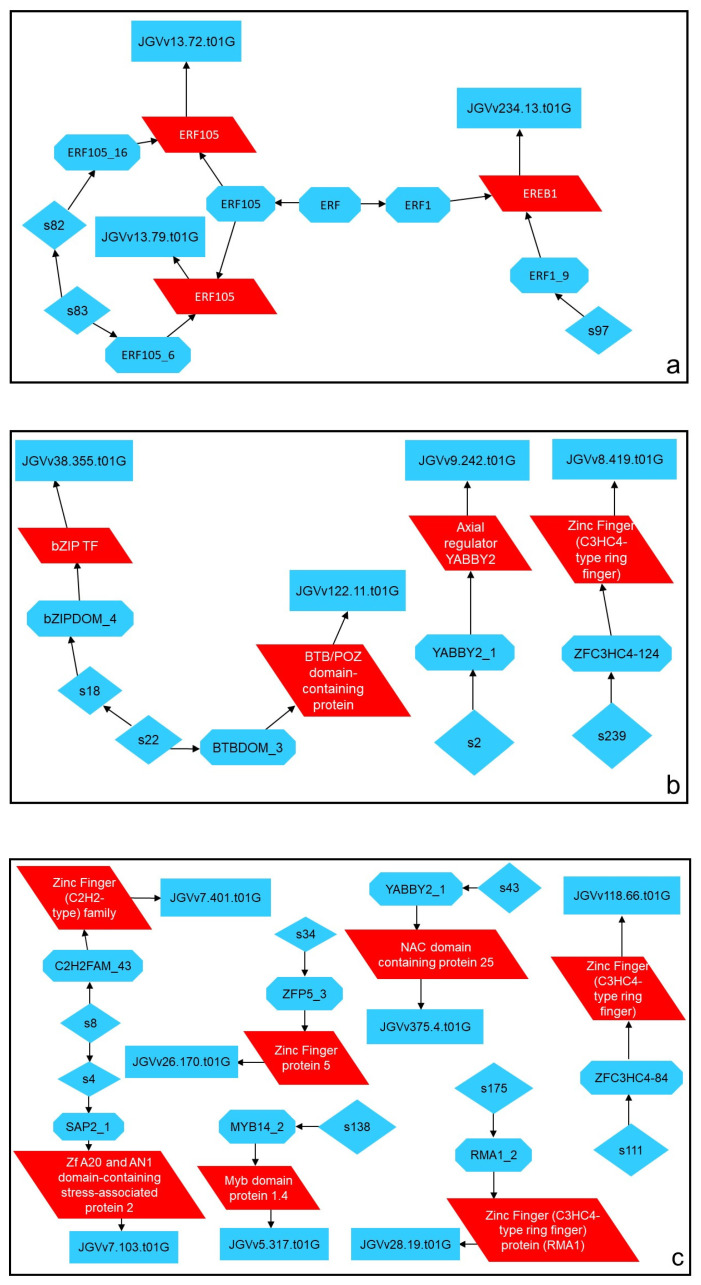
Cytoscape analysis of significantly up- or downregulated ethylene response factors (**a**) and transcription factors of interest (**b**,**c**) in regulated deficit irrigation (RDI); studied through RT-qPCR. The DEGs monitored in this project are highlighted in red. Rectangles represent genes, parallelograms represent RNAs, octagons represent proteins, and diamonds represent ions. This network was obtained by selecting our transcripts in the VitisNET network and selecting its first neighbors and its directed outgoing nodes.

In Figure 7a it is possible to visualize a sub-network created from the ethylene signaling network that shows the interactions between three DEGs assessed through RT-qPCR, *ERF105A*, *ERF105B*, and *EREB*. This network, alongside several other signaling networks, is responsible for the environmental processing information. Networks visualized in Figure 7b,c show the interactions between several transcription factors (TFs) that were significantly down- and upregulated, respectively. These two sub-networks were created from a general TFs network and show that most of these TFs do not have the same interactions that can be seen in Figure 7a. These combined results suggest that the different TFs have alternative pathways in regulating transcription, and that if there is any type of interaction between them, it may occur further downstream.

From all the DEGs, *APX1* (*Ascorbate peroxidase 1*) was the only gene that showed almost no changes in expression in pea size and *véraison* but was highly upregulated in full maturation (Figure 6), with the highest level of expression of all DEGs studied. APX1 has a paramount role in the protection of organelles against oxidative stress [45]. The expression of *APX1* in maturation indicates that it is contributing to the regulation of H_2_O_2_ levels, thus preventing excessive ROS accumulation during berry ripening, and preventing oxidative damage.

When comparing the expression of the selected DEGs during berry development in SDI (Figure 6b) and in RDI (Figure 6c), it was possible to see that in SDI most DEGs were downregulated in *véraison* and maturation. However, in RDI, although downregulated in *véraison*, most DEGs were upregulated in maturation. *HSP22*, *HSP23.6*, and *ERD4* were the only DEGs upregulated in both developmental stages. With an opposite response to water stress, *HSP17.8A* and *HSP17.8B* were upregulated in SDI in *véraison* and downregulated in maturation, but in RDI they were downregulated in *véraison* and upregulated in maturation. In the ERF family, *ERF105*, *EREB1*, and *ERF025-like* maintained the same pattern of downregulation in both conditions. Besides these genes, *HSP17.9A*, *HSP17.9B*, *HSP18.2B*, and *APX1* were also significantly downregulated in maturation SDI and significantly upregulated in RDI.

From the analysis of these regulatory patterns, it becomes apparent that a clear upregulation of the expression of key genes upon water stress was only achieved at maturation, when plants had been submitted to severe water stress from shortly before *véraison* (Figure S3). Touriga Nacional is a variety that withstands severe water stress without significant increase of global gene expression in leaves [9], which can be explained by the high levels of antioxidative metabolites found in those leaves, even under control conditions [35]. In berries, however, when drought becomes severe, several mechanisms of response to stress are activated, such as *sHSP*, *ERF,* and *APX* expression.

Gene expression during berry development was also modulated by the plant’s water status; in SDI there was a clear downregulation of the stress response genes studied in *véraison* and maturation with the exception of *HSP22, HSP23.6,* and *HSP26.5*. Under severe water stress (RDI in M), there was a significant upregulation of the genes studied, with the exception of *ERFs*, which were neither up- nor downregulated. In fact, the expression of the ERFs studied was repressed during development in non-stressful conditions. Therefore, in TN berry skins, and contrary to leaves, where ABA is the tolerance conferring hormone [19], the modulation of ethylene regulatory pathway genes points to a challenging tissue specific response. Conversely, *APX.1*, a gene coding a ROS-scavenging enzyme, and several genes coding sHSPs followed a similar trend as in TN leaves under abiotic stress [19], pointing to a general effect that is organ-independent. 

## 3. Materials and Methods

### 3.1. Field Conditions and Sampling 

The biological material was sampled in the season of 2015 in a commercial vineyard located at Herdade do Esporão, Reguengos de Monsaraz (38°23′42.0″ N, 7°32′51.4″ W). The vineyard has a planting density of 2200 vines per hectare, spaced 1.5 m within and 3.0 m between north–south oriented rows. Vines were trained on a vertical shoot positioning system with one pair of movable wires and spur-pruned on a bilateral Royat Cordon system with 16 nodes per vine. The experimental layout was a randomized complete block design with two deficit irrigation treatments: sustainable deficit irrigation (SDI—control used by the farm; 36% ETc) and regulated deficit irrigation (RDI; 24% ETc), and four replications per treatment. The elemental plot comprises three adjacent rows (two buffer rows and a central one for data collection). 

Weather conditions monitored during the season and pre-dawn leaf water potential also quantified during the season in both treatments are shown on Figure S3. Berries of *Vitis vinifera* L., var. Touriga Nacional (TN) were sampled at three different phenological stages in order to compare responses to drought in key stages of berry development: pea size (BBCH stage 75, G), *véraison* (BBCH stage 81, V), and full maturation (BBCH stage 85−89, M) berries. For each irrigation strategy and berry development, a pooled sample of circa 30 individuals, comprising three berries from the top, three from the middle, and three from the bottom sections of each cluster, was obtained. The berries were transported on ice to the lab, where the skin from 40 berries per pool was removed, reduced to powder in liquid nitrogen, and kept at −80 °C until further analysis. The pooling was performed in triplicate. RNA-Seq was performed on V and M samples, while RT-qPCR was performed on all samples.

### 3.2. RNA Extraction, Quantification and Quality Evaluation

RNA extraction was performed with Spectrum Plant Total RNA kit (Sigma-Aldrich, St. Louis, MO, USA), according to the manufacturer’s instructions, using 200 mg of powder for each sample. After the extraction, RNA samples were treated with RNase-free DNase I (Qiagen, Hilden, Germany) according to the manufacturer’s protocol. RNA was quantified through spectrophotometry in a Synergy HT Multiplate Reader (Biotek, Friedrichshall, Germany), using a Take3 Multi-Volume Plate (Biotek), with Gene5 software (Biotek). Sample quality was assessed through the A_260_/A_280_ ratio. Only samples with a ratio between 1.8 and 2.1 were chosen and submitted to RNA-sequencing (RNA-seq).

### 3.3. Transcriptome Sequencing and Mapping

Sequencing was performed in the Genomics and Transcriptomics Platform, University of Torino, Italy [46] Illumina RNA-Seq libraries were obtained from poly-(A) mRNA isolated from 2.5 μg aliquots. Libraries were sequenced with an Illumina HiSeq 1000 sequencer (Illumina, San Diego, CA, USA) and 75 bp single-ended sequences (Illumina) were generated. Preprocessing of low-quality reads (>50 bases with quality <7 or >10% undetermined bases) and putative PCR duplicate reads were removed, and Illumina TruSeq (Illumina) adapter sequences were clipped. Raw data reads from the RNA-Seq were trimmed using SeqtrimNEXT [47,48]. Trimming was set to remove indeterminations, poly-A-tails, Illumina adapters, contaminant sequences, vector residues, low-quality zones, and repetitive/complex regions. Transcriptome and annotation versions 12X_v2.1 of *Vitis vinifera vinifera* cv. PN40024 were downloaded from Grape Genome Database on CRIBI [49].

### 3.4. Gene Expression and Validation

The number of reads per kilobase transcript per million reads (RPKM) was quantified (Equation (1)) and assumed as an expression value [50].

(1) $$RPKM=\frac{number\;of\;reads\;covering\;the\;region}{\frac{total\;library\;reads}{1000000}\times \frac{region\;length\;\left(bp\right)}{1000}}$$

RPKMs were calculated individually for each sample, matching the cleaned reads library from each sample to the reference transcriptome. This assessment was achieved using RSEM package [51]. The reference transcriptome [49] was formatted using rsem-prepare-reference algorithm (with—bowtie option), and quantification was performed with rsem-calculate-expression, with—bowtie-n 3, to allow a mismatch of three nucleotides. Gene names and functions were assessed according to the publicly available annotation [49]. 

### 3.5. Bioinformatics Analysis 

The subsequent analyses were performed using RStudio R i386 3.3.2 [52]. The script used is available in the Supplementary Material (Supplementary File S1). In order to improve the quality of the assembly, transcripts below a threshold of ≥150 bp were discarded [53]. Only transcripts with RPKM ≥ 1 were considered as active transcripts [54].

#### Bioinformatics Validation and Overview 

To perform a bioinformatics validation, the function *cor* (method = “pearson”, use = “pairwise.complete.obs”) was used. In order to improve the visualization of the correlation between biological replicates. Natural logarithm was applied to the obtained RPKMs, and the correlation was assessed again, as described. A plot for each sample was drawn using the function *ggplot* from the package *ggplot2* (2.2.1) [55]. Linear regression was traced using the function *geo_smooth*, from *ggplot2*. Since both correlations, normal (data not shown) and natural logarithm, were strong, the data obtained from both replicates were merged and the average of the replicates was used for further analysis.

To verify how many isoforms were being expressed in each functional category, the transcripts were organized according to their functional category. In order to visualize these data, a figure with the number of active genes per functional category was created, using the function *ggplot* (ggplot2 package).

### 3.6. Differentially Expressed Genes

To evaluate which genes were putatively responding to the different water irrigation strategies (RDI and SDI), a ratio (Equation (2)) was calculated, considering SDI as control: for each transcript, the RPKM obtained using RDI was divided by the RPKM measured on SDI, on the equivalent maturation state.

(2) $$Ratio_{eq\left(2\right)}=\frac{RPKM_{RDI}\;}{RPKM_{SDI}}$$

When using this equation, the ratio of all downregulated genes will be a value between 0 and 1, and all the upregulated genes will have a ratio higher than 1. As these scales may lead to wrong interpretations, the ratio of the downregulated genes was inverted (Equation (3)) and therefore became a value lower than −1.

(3) $$Ratio_{down-regulated}=\frac{-1}{Ratio_{eq\left(2\right)}}$$

For further analysis, a cut off value of 1.5 was chosen, that is, ratios of which values were |>−1.5| were considered as significantly regulated in relation to the control. To verify the ratio distribution, the number of transcripts was calculated between intervals. A bar plot was created using the function *gap.barplot* from the package *plotrix* (3.6−5), with the argument gap = c() (specific intervals used, detailed in Supplemental File S1) [56]. 

The transcripts were ranked according to their ratio values, and the ten most upregulated and the ten most downregulated transcripts were determined for each sample and subjected to extend bioinformatics studies. The transcript sequences were obtained from the Grape Genome Database on CRIBI [49] by matching the transcript ID. The nucleotide sequences were submitted to a BLASTn [57] on TAIR, the Arabidopsis Thaliana Genome Database [58]), since this database has a more detailed information than CRIBI [49].

### 3.7. Sample-Specific Transcripts

To determine which transcripts were exclusively active in each sample, Venn diagrams were constructed, using the function *venn.diagram* from the R package VennDiagram (1.6.17) [59]. To select the transcripts exclusive to each sample, those present only on each condition (RM, SM, RV, and SV) were selected. The argument scaled = FALSE was applied to avoid proportional circles. Five Venn diagrams were constructed (i) to compare between the two irrigation strategies in the samples (RM vs. SM samples), (ii) to compare all samples, and (iii) to compare genes exclusively expressed in RM (RPKM > 1) or in SM (RPKM ≤ 1).

### 3.8. Correlation between RNA-Seq Results and Leaf Water Potential

To evaluate if the modulation of gene expression observed through RNA-Seq was effectively a response to the water stress conditions, leaf water potential at four times of the day (8 h, 11 h, 14 h, 17 h, and 19 h) was measured. The measurements were performed on 8 July for *véraison* and 14 August for full maturation berries. A total of four biological replicates were carried out at each time point. To correlate water potential (Ψ) with gene expression values from the whole sample set, the amplitudes (ΔΨ) of leaf Ψ were calculated (Equation (4)) and correlated with gene expression values from each transcript of the two biological replicates. This correlation was obtained using the function cor, considering a linear model. The coefficient of determination (R^2^) was calculated.

(4) ΔΨ=max (Ψ)−min(Ψ)

A threshold of R^2^ ≥ 0.99 was set. The sequences of the correlated transcripts that were not annotated in CRIBI were obtained by submitting the nucleotide sequences to a BLASTn [57] on TAIR [58] to infer their putative function.

### 3.9. Validation of Expression Profiles through Real Time Quantitative PCR (RT-qPCR)

cDNA was prepared using RevertAid Reverse Transcriptase kit (Thermo Fisher Scientific, Waltham, MA, USA) and oligo-dT primers (STABvida, Caparica, Portugal), according to the manufacturer’s instructions, using 1 µg RNA. Obtained cDNA was stored in −20 °C for further analysis.

Significantly expressed genes within functional categories of interest, such as response to stress, hormones, signaling, metabolism, and regulation, were selected. Primers were designed with Beacon Designer 4 (PREMIER Biosoft, Davis, CA, USA) software. Some sequences had already been found as significantly regulated in previous experiments on abiotic stress in leaves [8,9], and the primers designed for those experiments were used here (primer list, sequences, and amplification temperatures in Supplementary Table S3). RT-qPCR was performed in 96-well transparent reaction plates, using an iQ5 Real Time PCR (Bio-Rad, Hercules, CA, USA). Two biological replicates and two technical replicates were performed. The mix was composed of EvaGreen Master Mix (SsoFast_EvaGreen Supermix, Bio-Rad), prepared according to the manufacturer’s instructions, 10 μM of primer, diluted cDNA, and Milli-Q H_2_O to obtain a final volume of 20 µL. Amplification of PCR products was monitored with EvaGreen which is present in the Master Mix. The reactions consisted of an initial denaturation step at 95 °C for 2 min, with 50 cycles as follows: denaturation at 95 °C, annealing at the optimal temperature for each primer for 30 s, and elongation at 72 °C for 45 s, with a reading of fluorescence emission in the end of each cycle, followed by a final elongation at 72 °C for 5 min. cDNA concentration in the mix varied for 1 ng µL^−1^ to 15 ng µL^−1^, depending on the sample, and C_q_s were adjusted so that quantifications were performed homogenously.

Data were collected between cycles 5 and 17 in order to obtain a baseline subtracted logarithmic amplification plot of the fluorescence signal (ΔRn). The *R_n_* threshold was defined as 50 so as to obtain C_q_ values in the beginning of the exponential amplification. Values were further exported to Excel, and quantification of the relative gene expression was achieved with the ΔΔC_q_ method [60]. Three genes were used as reference, *Actin 2* (*ACT*), *Vitis vinifera translation initiation factor 3 subunit G* (*TIF*), and *Vitis vinifera translation initiation factor eIF-2B subunit alpha* (*TIF-GTP*). These genes were shown to be stable under conditions of abiotic stress, namely, water stress [61].

### 3.10. Statistical Analysis of RT-qPCR Results

Significant variations in the expression of the selected genes between SDI and RDI were considered when |log_2_ (gene expression level)| > 2. The same process was applied to the comparison during berry development, using pea size as the control. Variations between developmental stages in RDI and between RDI and SDI in each developmental stage were identified with Student *t*-tests (Excel, Microsoft, Albuquerque, NM, USA), and were considered significant when *p*-value < 0.05. 

## 4. Conclusions

In the present work, a comprehensive analysis of a RNA-Seq array from the berry skin of Touriga Nacional at *véraison* and full maturation under two levels of deficit irrigation, SDI and RDI, provided a list of candidate transcripts that are potentially responding to water stress. It was possible to conclude that the developmental stage affects the global response of the berry transcriptome to water deficit. At full maturation, berries had fewer transcriptional changes upon water stress compared to *véraison*. 

The validation by RT-qPCR of the data collected by RNA-Seq of samples from the same experiment but at three developmental stages, pea size, *véraison,* and full maturation, allowed a robust and comprehensive assay of the function of the DEGs that significantly responded to water stress, and their function in berry skin development and protection. The analysis of the regulatory patterns indicated an activation of mechanisms of response to stress, such as *sHSP*, *ERF*, and *APX*. Although an upregulation response was common to all, there were differences in regulation, with *ERFs* and *APX1* showing very low levels of expression under non-stress conditions and *sHSPs* strongly upregulated upon stress. This pattern of response in TN berry skin was unlike the one reported in leaves [19] and highlights the organ specificity of the stress response. 

It is worth highlighting that the majority of the transcripts that correlate with water stress belonged to the functional category “metabolism”, responsible for changes in fundamental metabolic pathways associated with berry development. Some of the typical wine characteristics derive from the presence of secondary metabolites produced in the berry, specifically in the berry skin, hence the importance of further studies addressing the metabolic alterations that water stress could lead to.

## Figures and Tables

**Table 1:** Correlation between biological replicates. Irrigation strategies are RDI (regulated deficit irrigation) and SDI (sustained deficit irrigation). Developmental stages are *véraison* and full maturation; r represents the coefficient of correlation between replicates. Natural logarithm was applied to the original RPKM values. All samples had a *p*-value lower than 2.2 × 10^−16^.

Irrigation Strategy	Phenological Stage	R
SDI	*Véraison*	0.984
	Maturation	0.989
RDI	*Véraison*	0.989
	Maturation	0.974

**Table 2:** Functional profiling of the expression of all non-annotated transcript categories for each RNA-Seq library according to each transcript functional category. The number of transcripts (length ≥ 150 and RPKM ≥ 1) per functional category was divided by the total transcripts from that category in order to normalize the expression profiles according to the number of transcripts on each class. SV: SDI *véraison*; RV: RDI *véraison*; SM: SDI full maturation; RM: RDI full maturation.

%	SV	RV	SM	RM
Unknown	47	46	45	45
Unclear	43	42	41	40
Unclassified	45	44	42	41
No Name	27	27	26	26
No Hit	26	25	22	23

**Table 3:** Detailed analysis of the transcripts up- (↑) and downregulated (↓) in the functional categories signaling and RT—transcription factors, according to the results shown in Figure 4. “Pathway” in signaling is represented by calcium sensors and signaling, protein kinases, protein phosphatases, and signaling molecules.

	Signaling								RT—Transcription Factors									
	Pathway	ABA	Auxin	Brassinosteroid	Cytokinin	Ethylene	Gibberellic Acid	Jasmonate Salicylate	bZIP	C2C2-YABBY	C2H2	Homeobox Domain	MYB	NAC	TRAF	Zinc Finger AN1	Zinc Finger C3HC4	
RDI	↑	6	-	2	-	-	1	1	1	-	-	2	-	1	1	-	1	2
	↓	4	-	7	-	-	2	-	-	1	1	-	-	-	-	1	-	1
SDI	↑	3	-	1	1	-	1	-	1	-	-	3	-	-	2	-	-	3
	↓	12	2	5	-	1	3	1	-	-	-	-	1	-	-	-	-	-

**Table 4:** Detailed analysis of the transcripts up- (↑) and downregulated (↓) in the functional categories signaling and RT—transcription factors according to the results showed in Figure 4. “Pathway” in signaling is only represented by protein kinases.

		Signaling				RT—Transcription Factors		
		Pathway	Auxin	Ethylene	Jasmonate Salicylate	bZIP	WRKY	Zinc Finger C3HC4
V	↑	-	1	-	1	1	-	-
	↓	2	2	3	-	-	1	1
M	↑	-	-	-	-	-	-	-
	↓	-	-	-	-	-	-	-

## Data Availability

Not applicable.

## Supplementary Materials

The following supporting information can be downloaded at: https://www.mdpi.com/article/10.3390/plants11060827/s1, File S1: R Script; Figure S1: Correlation between biological replicates in RNA-Seq; Figure S2: Functional profiling of expression of non-annotated transcript categories for each RNA-Seq library; Figure S3: Monthly maximum, average, and minimum temperatures, total monthly precipitation, and pre-dawn leaf water potential (Ψpd) during the growing season; Table S1: Correlation of transcription with pre-dawn leaf water potential (Ψpd); Table S2: Top ten up- and downregulated DEGs in *véraison* and maturation; Table S3: List of primers utilized in RT-qPCR.

## References

[1] (2021). OIV, State of the Vitivinicultural Sector in 2020. https://www.oiv.int/public/medias/7909/oiv-state-of-the-world-vitivinicultural-sector-in-2020.pdf.

[2] Fraga H. (2020). Climate Change: A New Challenge for the Winemaking Sector. Agronomy.

[3] Carvalho L.C., Amâncio S. (2018). Cutting the Gordian Knot of abiotic stress in grapevine: From the test tube to climate change adaptation. Physiol. Plant.

[4] Giorgi F., Lionello P. (2008). Climate change projections for the Mediterranean region. Glob. Planet Change.

[5] De Orduña R.M. (2010). Climate change associated effects on grape and wine quality and production. Food Res. Int..

[6] Olesen J.E., Trnka M., Kersebaum K.C., Skjelvåg A.O., Seguin B., Peltonen-Sainio P., Rossi F., Kozyra J., Micale F. (2011). Impacts and adaptation of European crop production systems to climate change. Eur. J. Agron..

[7] Dal Santo S., Zenoni S., Sandri M., De Lorenzis G., Magris G., De Paoli E., Di Gaspero G., Del Fabbro C., Morgante M. (2018). Grapevine field experiments reveal the contribution of genotype, the influence of environment and the effect of their interaction (G×E) on the berry transcriptome. Plant J..

[8] Carvalho L.C., Silva M., Coito J.L., Rocheta M.P., Amâncio S. (2017). Design of a custom RT-qPCR array for assignment of abiotic stress tolerance in traditional Portuguese grapevine varieties. Front Plant Sci..

[9] Rocheta M., Coito J.L., Ramos M.J.N., Carvalho L.C., Becker J.D., Carbonell-Bejerano P., Amâncio S. (2016). Transcriptomic comparison between two *Vitis vinifera* L. varieties (Trincadeira and Touriga Nacional) in abiotic stress conditions. BMC Plant Biol..

[10] Girona J., Mata M., del Campo J., Arbonés A., Bartra E., Marsal J. (2006). The use of midday leaf water potential for scheduling deficit irrigation in vineyards. Irrig. Sci..

[11] Girona J., Marsal J., Mata M., Del Campo J., Basile B. (2009). Phenological sensitivity of berry growth and composition of tempranillo grapevines (*Vitis Vinifera* L.) to water stress. Aust. J. Grape Wine Res..

[12] Chaves M.M., Zarrouk O., Francisco R., Costa J.M., Santos T., Regalado A.P., Rodrigues M.L., Lopes C.M. (2010). Grapevine under deficit irrigation: Hints from physiological and molecular data. Ann. Bot..

[13] Deytieux-Belleau C., Geny L., Roudet J., Maet V., Donèchoe B., Fermaud M. (2009). Grape berry skin features related to ontogenic resistance to *Botrytis cinerea*. Eur. J. Plant Pathol..

[14] Carbonell-Bejerano P., Carvalho L.C., Dias J.E.E., Martinez-Zapater J.M., Amâncio S. (2015). Exploiting *Vitis* genetic diversity to manage with stress. Grapevine in a Changing Environment: A Molecular and Ecophysiological Perspective.

[15] Savoi S., Wong D.C.J., Arapitsas P., Miculan M., Bucchetti B., Peterlunger E., Fait A., Mattivi F., Castellarin S.D. (2016). Transcriptome and metabolite profiling reveals that prolonged drought modulates the phenylpropanoid and terpenoid pathway in white grapes (*Vitis vinifera* L.). BMC Plant Biol..

[16] Castellarin S.D., Matthews M.A., Di Gaspero G., Gambetta G.A. (2007). Water deficits accelerate ripening and induce changes in gene expression regulating flavonoid biosynthesis in grape berries. Planta.

[17] Finotello F., Di Camillo B. (2015). Measuring differential gene expression with RNA-seq: Challenges and strategies for data analysis. Brief. Funct. Genom..

[18] Palumbo M.C., Zenoni S., Fasoli M., Massonnet M., Farina L., Castiglione F., Pezzotti M., Paci P. (2014). Integrated Network Analysis Identifies Fight-Club Nodes as a Class of Hubs Encompassing Key Putative Switch Genes That Induce Major Transcriptome Reprogramming during Grapevine Development. Plant Cell.

[19] Carvalho L.C., Coito J.L., Colaço S., Sangiogo M., Amâncio S. (2015). Heat stress in grapevine: The pros and cons of acclimation. Plant Cell Environ..

[20] Carvalho L.C., Coito J.L., Gonçalves E.F., Chaves M.M., Amâncio S. (2016). Differential physiological response of the grapevine varieties Touriga Nacional and Trincadeira to combined heat, drought and light stresses. Plant Biol..

[21] Hochberg U., Degu A., Toubiana D., Gendler T., Nikoloski Z., Rachmilevitch S., Fait A. (2013). Metabolite profiling and network analysis reveal coordinated changes in grapevine water stress response. BMC Plant Biol..

[22] Griesser M., Weingart G., Schoedl-Hummel K., Neumann N., Becker M., Varmuza K., Liebner F., Schuhmacher R., Forneck A. (2015). Severe drought stress is affecting selected primary metabolites, polyphenols, and volatile metabolites in grapevine leaves (*Vitis vinifera* cv. Pinot noir). Plant Physiol. Biochem..

[23] Santner A., Estelle M. (2009). Recent advances and emerging trends in plant hormone signalling. Nature.

[24] Argueso C.T., Ferreira F.J., Kieber J.J. (2009). Environmental perception avenues: The interaction of cytokinin and environmental response pathways. Plant Cell Environ..

[25] Zhang S.W., Li C.H., Cao J., Zhang Y.C., Zhang S.Q., Xia Y.F., Sun D.Y., Sun Y. (2009). Altered Architecture and Enhanced Drought Tolerance in Rice via the Down-Regulation of Indole-3-Acetic Acid by TLD1/OsGH3.13 Activation. Plant Physiol..

[26] Shinozaki K., Yamaguchi-Shinozaki K. (2007). Gene networks involved in drought stress response and tolerance. J. Exp. Bot..

[27] Santana-Vieira D.D., Freschi L., Almeida L.A., Moraes D.H., Neves D.M., Santos L.M., Bertolde F.Z., Filho W.S.S., Filho M.A.C., Gesteira A.S. (2016). Survival strategies of *citrus* rootstocks subjected to drought. Plant Cell Physiol..

[28] Gimeno J., Gadea J., Forment J., Pérez-Valle J., Santiago J., Martínez-Godoy M.A., Yenush L., Bellés J.M., Brumós J., Colmenero-Flores J.M. (2009). Shared and novel molecular responses of mandarin to drought. Plant Mol. Biol..

[29] Huang X.S., Liu J.H., Chen X.J. (2010). Overexpression of *PtrABF* gene; a bZIP transcription factor isolated from *Poncirus trifoliate*, enhances dehydration and drought tolerance in tobacco via scavenging ROS and modulating expression of stress-responsive genes. BMC Plant Biol..

[30] Muthamilarasan M., Bonthala V.S., Mishra A.K., Khandelwal R., Khan Y., Roy R., Manoj P. (2014). C2H2 type of zinc finger transcription factors in foxtail millet define response to abiotic stresses. Funct. Integr. Genom..

[31] Jung Y.Y., Lee I.H., Nou I.S., Lee K.D., Rashotte A.M., Kang K.K. (2013). BrRZFP1 a *Brassica rapa* C3HC4-type RING zinc finger protein involved in cold, salt and dehydration stress. Plant Biol..

[32] Carvalho L.C., Vilela B.J., Mullineaux P.M., Amâncio S. (2011). Comparative Transcriptomic Profiling of *Vitis vinifera* Under High Light Using a Custom-Made Array and the Affymetrix GeneChip. Mol. Plant.

[33] Wang W., Vinocur B., Altman A. (2003). Plant responses to drought, salinity and extreme temperatures: Towards genetic engineering for stress tolerance. Planta.

[34] Rocheta M., Becker J.D., Coito J.L., Carvalho L.C., Amâncio S. (2014). Heat and water stress induce unique transcriptional signatures of heat-shock proteins and transcription factors in grapevine. Funct. Integr. Genom..

[35] Barua D., Downs C., Geckathorn S. (2003). Variation in chloroplast small heat-shock protein function is a major determinant of variation in thermotolerance of photosynthetic electron transport among ecotypes of *Chenopodium album*. Func. Plant Biol..

[36] Punja Z.K., Zhang Y.Y. (1993). Plant chitinases and their roles in resistance to fungal diseases. J. Nematol..

[37] Wang N., Zheng Y., Xin H., Fang L., Li S. (2013). Comprehensive analysis of NAC domain transcription factor gene family in *Vitis vinifera*. Plant Cell Rep..

[38] Yang Y., Wang Y., Jia L., Yang G., Xu X., Zhai H., He H., Li J., Dai X., Qin N. (2018). Involvement of an ABI-like protein and a Ca^2+^-ATPase in drought tolerance as revealed by transcript profiling of a sweet potato somatic hybrid and its parents *Ipomoea batatas* (L.) Lam. and *I. triloba* L. PLoS ONE.

[39] Pastori G.M., Foyer C.H. (2002). Common components, networks, and pathways of cross-tolerance to stress: The central role of ‘redox’ and abscisic acid-mediated controls. Plant Physiol..

[40] Cheng M.C., Liao P.M., Kuo W.W., Lin T.P. (2013). The *Arabidopsis* Ethylene Response Factor1 regulates abiotic stress-responsive gene expression by binding to different cis-acting elements in response to different stress signals. Plant Physiol..

[41] Müller M., Munné-Bosch S. (2015). Ethylene Response Factors: A Key Regulatory Hub in Hormone and Stress Signaling. Plant Physiol..

[42] Dubois M., Skirycz A., Claeys H., Maleux K., Dhondt S., De Bodt S., Bossche R.V., De Milde L., Yoshizumi T., Matsui M. (2013). Ethylene Response Factor6 acts as a central regulator of leaf growth under water limiting conditions in *Arabidopsis*. Plant Physiol..

[43] Meng X., Xu J., He Y., Yang K.Y., Mordorski B., Liu Y., Zhang S. (2013). Phosphorylation of an ERF transcription factor by *Arabidopsis* MPK3/ MPK6 regulates plant defense gene induction and fungal resistance. Plant Cell.

[44] Sewelam N., Kazan K., Thomas-Hall S.R., Kidd B.N., Manners J.M., Schenk P.M. (2013). Ethylene response factor 6 is a regulator of reactive oxygen species signaling in *Arabidopsis*. PLoS ONE.

[45] Davletova S., Rizhsky L., Liang H., Shengqiang Z., Oliver D.J., Coutu J., Shulaev V., Schlauch K., Mittler R. (2005). Cytosolic Ascorbate Peroxidase 1 is a Central Component of the Reactive Oxygen Gene Network of *Arabidopsis*. Plant Cell.

[46] Genomics and Transcriptomics Platform. https://cpt.univr.it/en/genomics-and-transcriptomics-platform/.

[47] Falgueras J., Lara A.J., Fernandez-Pozo N., Canton F.R., Perez-Trabado G., Claros M.G. (2010). SeqTrim: A high-throughput pipeline for preprocessing any type of sequence reads. BMC Bioinform..

[48] SeqtrimNEXT. https://github.com/dariogf/SeqtrimNext.

[49] CRIBI. https://genomes.cribi.unipd.it/grape/.

[50] Mortazavi A., Williams B.A., McCue K., Schaeffer L., Wold B. (2008). Mapping and quantifying mammalian transcriptomes by RNA-Seq. Nat. Methods.

[51] Kelley J.L., Passow C.N., Plath M., Rodriguez L.A., Yee M.-C., Tobler M. (2012). Genomic resources for a model in adaptation and speciation research: Characterization of the *Poecilia mexicana* transcriptome. BMC Genom..

[52] RStudio Team (2020). RStudio: Integrated Development for R. RStudio.

[53] Chang Z., Wang Z., Li G. (2014). The impacts of read length and transcriptome complexity for *de novo* assembly: A simulation study. PLoS ONE.

[54] Ramos M.J.N., Coito J.L., Fino J., Cunha J., Silva H., de Almeida P.G., Costa M.M.R., Amâncio S., Paulo O.S., Rocheta M. (2017). Deep analysis of wild *Vitis* flower transcriptome reveals unexplored genome regions associated with sex specification. Plant Mol. Biol..

[55] Wickham H. (2016). ggplot2: Elegant Graphics for Data Analysis.

[56] Plotrix Package—Rdocumentation. https://www.rdocumentation.org/packages/plotrix/versions/3.7.

[57] Altschul S.F., Madden T.L., Schäffer A.A., Zhang J., Zhang Z., Miller W., Lipman D.J. (1997). Gapped BLAST and PSI-BLAST: A new generation of protein database search programs. Nuc. Acid Res..

[58] TAIR, the Arabidopsis Thaliana Genome Database. https://www.arabidopsis.org.

[59] Chen H., Boutros P.C. (2011). VennDiagram: A package for the generation of highly-customizable Venn and Euler diagrams in R. BMC Bioinform..

[60] Livak K.J., Schmittgen T.D. (2001). Analysis of relative gene expression data using real-time quantitative PCR and the 2^(−ΔΔC(T))^ Method. Methods.

[61] Coito J.L., Rocheta M., Carvalho L.C., Amâncio S. (2012). Microarray-based uncovering reference genes for quantitative real time PCR in grapevine under abiotic stress. BMC Res. Notes.

