# Supplementary material for: Developmental Regulation of Transcription in Touriga Nacional Berries under Deficit Irrigation
Source: Plants (Basel). 2022 Mar 21;11(6):827. doi: 10.3390/plants11060827 (PMC8955924; doi:10.3390/plants11060827)
Supplement: Supplementary file 1 [file plants-11-00827-s001.zip › plants-1619755-Supplementary.pdf]

Supplementary File S1. R script with all the instructions and commands used on R Studio along this project. Available on http://home.isa.utl.pt/~aa21133/supplemental_r_script.r

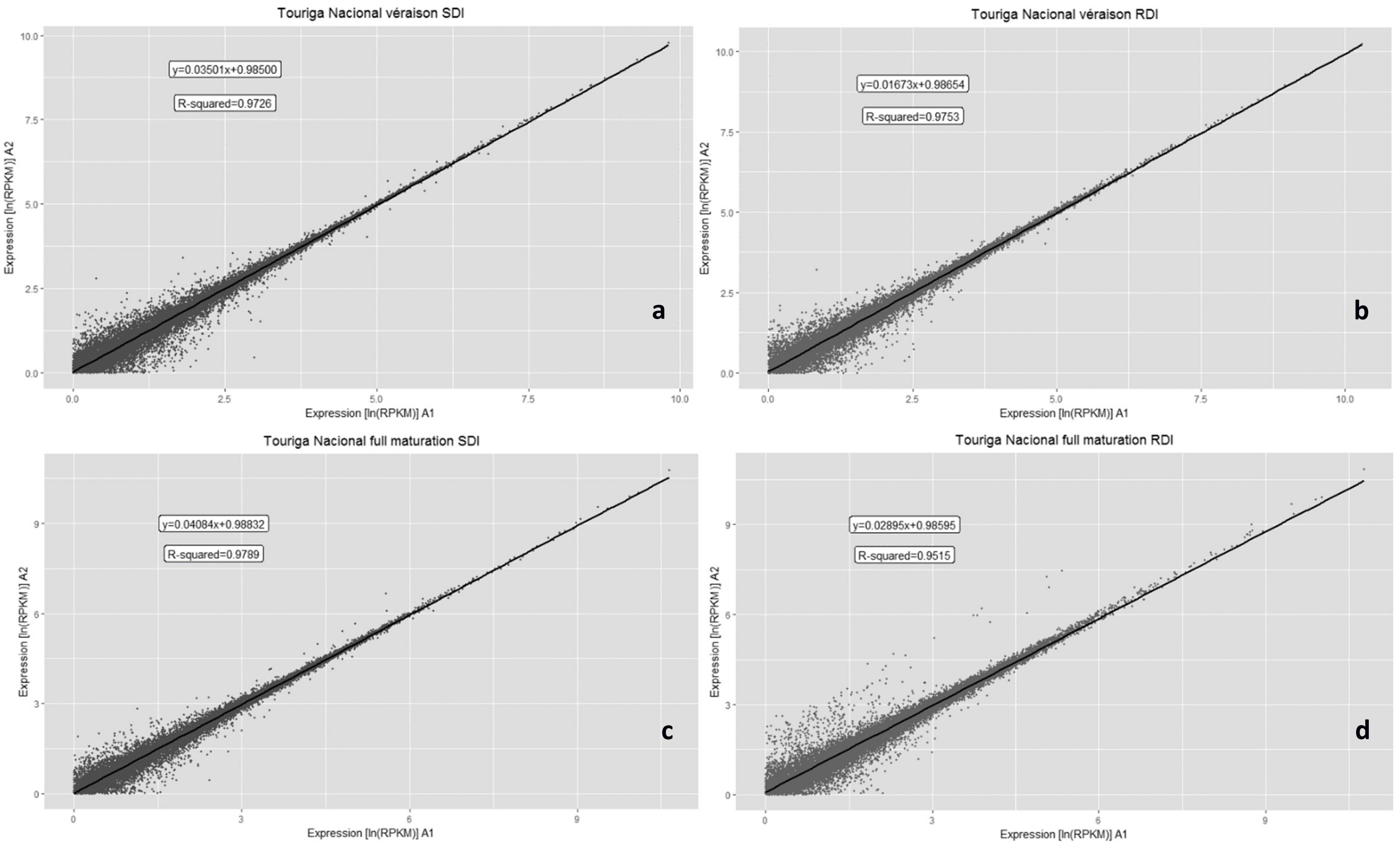

Supplemental Figure S1. Correlation between biological replicates in RNA-seq. Correlation between the logarithmized Reads per Kilobase transcript per Million reads (RPKMs) of two biological replicas (A1 and A2). Dots represents the transcripts. Pearson Method was used to calculate linear regression (black line). Coefficient of determination (R-squared) and the linear regression equation are provided; (**a**) *véraison* SDI; (**b**) *véraison* RDI; (**c**) full maturation SDI; (**d**) full maturation RDI.

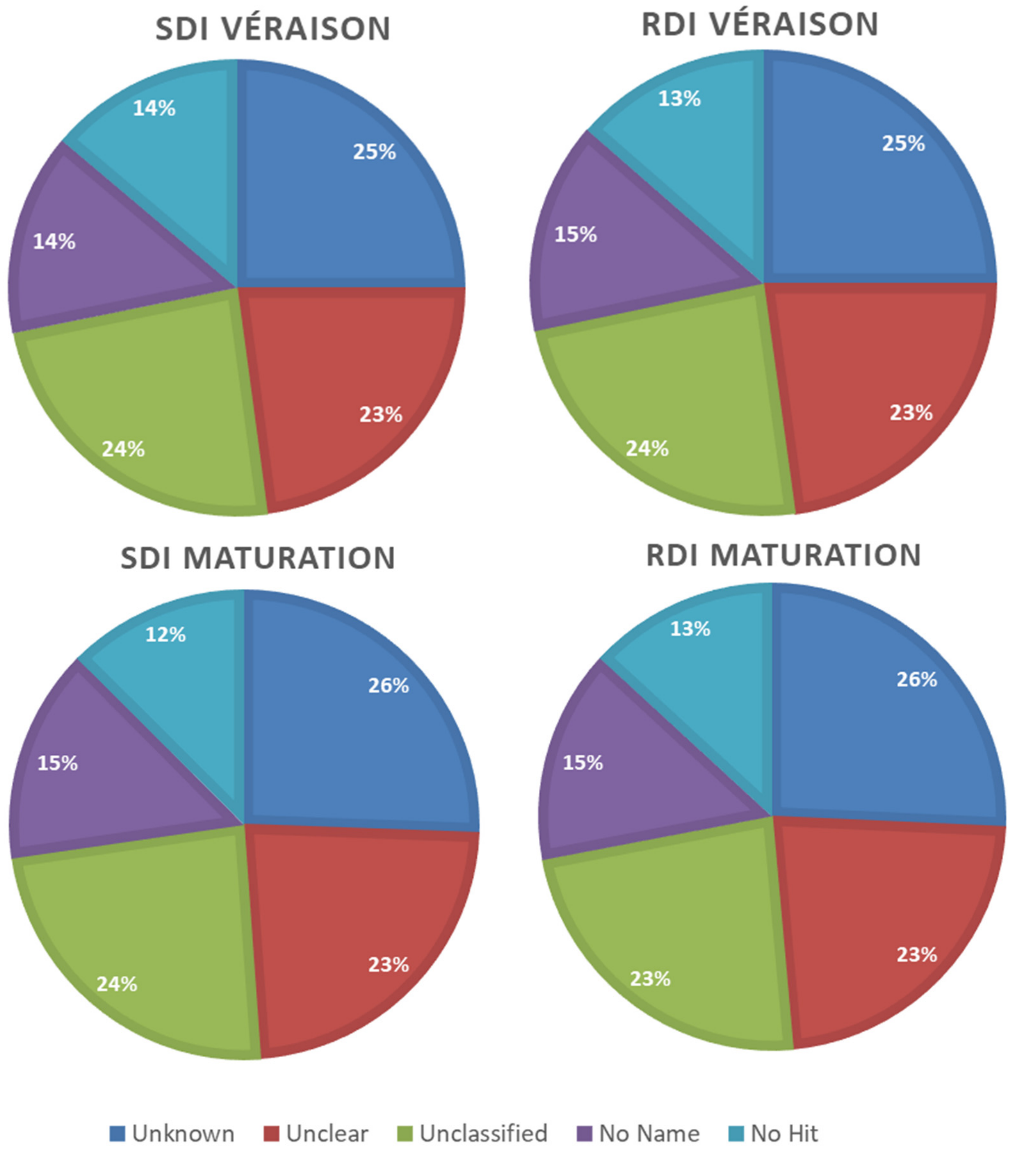

Supplemental Figure S2. Functional profiling of the expression of all non-annotated transcript categories for each RNA-Seq library, according to each transcript Functional Category. The number of expressed transcripts (length ≥ 150 and RPKM ≥ 1) per Functional Category, was divided by the total transcripts from that category, in order to normalize the expression profiles according to the number of transcripts on each class.

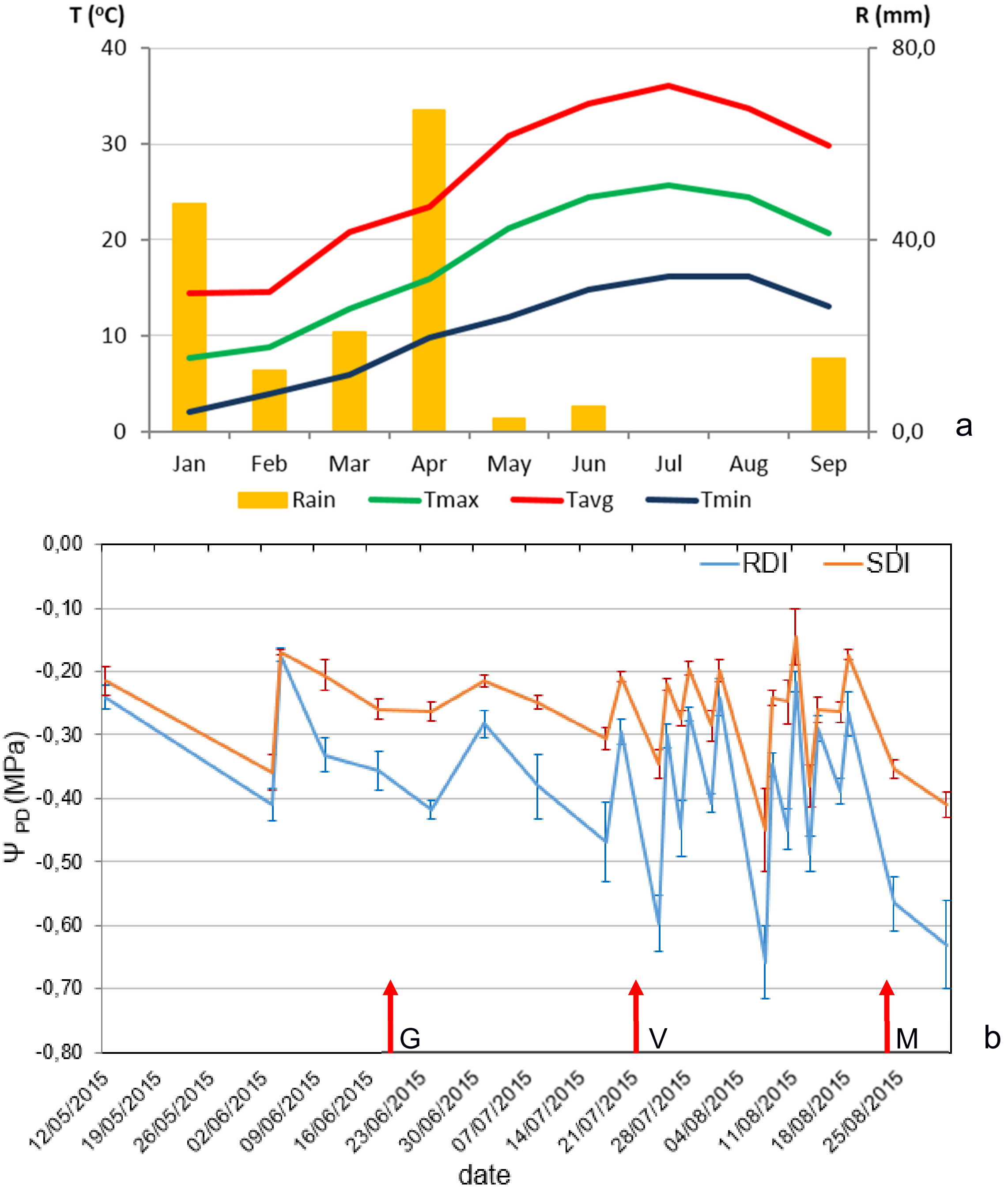

Supplemental Figure S3. Monthly maximum (max), average (avg) and minimum (min) temperatures and total monthly precipitation (a); leaf pre-dawn water potential (Ψ_{pd}) (b), measured with a pressure chamber, Model 600, PMS Instruments Company (Albany, OR) in Sustained Deficit Irrigation (SDI) and regulated Deficit Irrigation (RDI), plants at Esporão vineyard (38°23'42.0"N 7°32'51.4"W), throughout 2015. Sampling at pea size (G), *véraison* (V) and full maturation (R) indicated with red arrows.
